# Psychosocial risk and protective factors of secondary school dropout in Luxembourg: the protocol of an exploratory case-control study

**DOI:** 10.1186/1471-2458-11-555

**Published:** 2011-07-13

**Authors:** Pascale Esch, Valéry Bocquet, Charles Pull, Sophie Couffignal, Marc Graas, Marie-Lise Lair, Torsten Lehnert, Laurence Fond-Harmant, Marc Ansseau

**Affiliations:** 1 Centre for Health Studies, Centre de Recherche Public de la Santé, Rue Thomas Edison 1 A-B, 1445 Strassen, Luxembourg; 2 Department of clinical sciences, psychiatry and medical psychology, University of Liège, Avenue de l'Hôpital 13, 4000 Liège, Belgium; 3 Competence Centre of Methodology and Statistics, Centre de Recherche Public de la Santé, Rue Thomas Edison 1 A-B, 1445 Strassen, Luxembourg; 4 Laboratory of Emotional Disorders, Centre de Recherche Public de la Santé, Rue Thomas Edison 1 A-B, 1445 Strassen, Luxembourg; 5 Department of psychiatry, Centre Hospitalier du Kirchberg, Rue Edward Steichen 9, 2540 Luxembourg, Luxembourg

## Abstract

**Background:**

In Luxembourg, the extensive phenomenon of school dropout is a prime policy concern in the light of individual, social and economic consequences. Although the authorities report an overall decrease of the national dropout rate, the proportion of early school leavers who remain without any specific occupation is still alarming. Therefore, this study intends a shift of focus from system-inherent to individual factors, including mental health and family correlates, to provide a more comprehensive analysis of the dropout phenomenon.

**Methods/Design:**

The objectives of this study are to investigate the type and prevalence of psychiatric disorders among school dropouts and to compare the findings with those by a matched control group of regularly enrolled students. Furthermore, family variables and socioeconomic status will be analysed, as they are factors likely to interfere with both educational attainment and mental health. A trained psychologist will use structured interviews and self-report forms to investigate for mental health issues, information on schooling, socioeconomic situation and family life. Controls will be matched for gender, age, school type and educational grade.

**Discussion:**

As school dropouts face a serious risk of long term professional and social marginalization, there is an evident need for action. Identifying psychosocial risk and protective factors of school dropout will deliver solid insight on how to conceive public health strategies for young people who may need a more customized support to carry out their academic potential.

**Trial registration:**

ClinicalTrials.gov Identifier: NCT01354236

## Background

This research is part of an "Interreg IV-A" project aimed at analyzing and promoting mental health in the trans-border zone of Saarland (Germany), Lorraine (France), Wallonia (Belgium) and Luxembourg (PPSM "Prévention et Promotion de la Santé Mentale"[1]). The PPSM project consists of promoting mental health by developing knowledge on risk and resource factors, implementing campaigns to sensitise for overall well-being and establishing an international knowledge network of good practice in mental health. In Luxembourg, the evaluation and promotion of mental well-being will focus on adolescents and young adults who dropped out of school and who may be more at risk to have mental health issues.

Although school dropout is not a recent topic, yet it has never been as discussed than in today's knowledge based society. The eclecticism of definitions and studies concerning early school leaving well reflect the complexity of the subject. In this article, the terms "school dropout" and "early school leaving" will be used interchangeably.

Definitions account for national specificities such as the educational system policies and the duration of compulsory education. The Organisation for Economic Co-operation and Development (OECD) and EUROSTAT have developed indicators that allow to compare national dropout rates, however conclusions should consider limitations due to the national specificities. Both indicators define school dropout as the "termination of schooling below upper secondary qualification and the absence of any educational program or training". They slightly differ on the age span, which spreads from 18 to 24 years for EUROSTAT and only from 20 to 24 years for the OECD.

In Luxembourg, since 2003, the Ministry for Education ("Ministère de l'Eduction Nationale et de la Formation Professionnelle" MENFP) has been a third source providing information on secondary school dropout. In contrast to the OECD and EUROSTAT, the ministry records dropouts of all ages, reported by the national school institutions. For the scope of this research project, we will focus on the data released by the MENFP because it is based on actual figures.

Between 2003 and 2006, the national authorities had to face dropout rates of more than 15%. As a result, they introduced accompanying and preventive measures to reduce linguistic and cultural risk factors that may impede educational engagement and vocational decisions. Since then, the dropout rate has overall declined. According to the latest figures, the national dropout rate, for the academic year 2008/2009, was set at 9% [2] and hence conceded with the European benchmark of no more than 10%. However the proportion of early school leavers who remain without any specific occupation is still alarming.

When questioned on their rationale for leaving school, students reported 1) difficulties in vocational guidance, 2) academic failure or the anticipation of failure, 3) a lack of incentive to pursue any formal education, 4) the lack of an apprenticeship, 5) personal reasons, or 6) health issues [2]. In the light of these statements, dropout prevention needs to consider what may underlie the reported difficulties, by examining factors relating to the student. Insight into the correlates that can influence alienation from school, and thus dropout, will be provided by exploring individual factors such as mental health, family life and socioeconomic situation.

Adolescence can be an unsettling period of life, marked by physical, psychological and social changes. It is known that up to 50% of lifetime mental disorders have their onset during adolescence [3] and that prevalence rates of at least one DSM IV disorder range from 13.7% [4] to 22.2% [5]. Such disorders include substance use, disruptive behaviour disorders, anxiety and mood disorders.

Nevertheless, in Europe, the exploration of early school leaving in terms of mental health is yet to mature, and only a few recent studies can be referenced. In France, Huerre and Leroy [6] presented a holistic analysis of school dropout, Brandibas [7] considered truancy to be associated with different types of anxiety, whereas Legleye [8] reported an association between dropout and the progression to daily cannabis use. Jonsson [9] pointed out that Swedish adolescents suffering from depression were less likely than their non-depressed peers to have graduated from higher education.

In North American and Canadian research literature, several studies explored the impact of mental health factors on academic attainment. They covered a whole spectrum of psychiatric disorders, and concluded that school dropout is not necessarily associated with motivational or institutional factors, but with serious social and cognitive impairments aroused by mental illness. Meldrum [10] confirmed the above hypothesis by revealing that up to 15% of Canadian students interrupted their studies for reasons of mental health. A recent study of a US national sample confirmed that 12 out of 17 psychiatric disorders were associated with subsequent failure to complete secondary education by the age of 18 [11]. After controlling for potential confounders, the authors established that bipolar and conduct disorders were most consistently related to early school leaving. In fact, several studies concluded that there was a significant association between school dropout, Attention Deficit (Hyperactivity) Disorder/AD(H)D [12] and disruptive behaviour disorders, also known as externalizing disorders [13,14]. For Kessler [15], the association with externalizing disorders was significant for males only, whereas so-called internalizing disorders were the most important psychiatric determinants of school dropout for females. Internalizing disorders include anxiety (specific phobia, social phobia, generalized anxiety disorder, panic disorder with or without agoraphobia, obsessive compulsive disorder and posttraumatic stress disorder) and mood disorders (major depressive disorder, bipolar disorder, dysthymia). In 2008, Fletcher [16] confirmed these findings by detecting a significant negative correlation between depression and high school attainment for females only; whereas Jonsson [9] did not observe any difference in gender.

In addition to the disorder categories mentioned above, substance abuse and dependence have significantly correlated with school dropout [13,17,18]. In their literature review, Townsend et al. analyzed 46 studies on the correlation between substance use and school dropout, and concluded that alcohol, tobacco and cannabis use had an impact on educational attainment and vice versa [19].

Regarding the consensus for a multifactorial approach to psychiatric disorders, we consider it crucial to investigate for risk factors and protective factors derived from the individual and social domains as well as interactions among those factors. Socioeconomic status (SES) was reported to have an impact on both mental health [20] and educational attainment [21]. Indeed, among students with a low SES, 44% of school dropout could be attributed to psychiatric disorder, whereas the proportion increased to 61% for students with a high SES [22].

In the same way, family variables correlated with both mental health [23,24] and education [25,26]. In 1996, Bernstein and Borchardt analyzed different types of family composition in school refusal. They found out that communication and role behaviour seemed to be problematic in mother-only households [27]. According to Bernstein, difficulties in role behaviour may reflect symptoms of separation anxiety in children, who refuse to leave home because they worry about or care for a physically or emotionally vulnerable parent [23].

In the light of previous research, we hypothesize that school dropouts have significantly more mental health problems than their peers who go to school. They are also more likely to report a poor socioeconomic background, negative school experiences (repeating classes, disciplinary measures, absenteeism, problematic relations with authority and peers) and difficulties in family functioning (task fulfilment, role performance, communication, affective expression, connectedness, control behaviour, values and standards).

### Research questions

The first objective of this research is to assess the type and prevalence of psychiatric disorders among early school leavers in Luxembourg and to compare the findings with those by a control group matched for gender, age, school and educational grade. If there is an association between mental illness and school dropout in Luxembourg, is it significant for only one psychiatric disorder or more (comorbid disorders)?

As a second objective, we aim to identify specific protective and risk factors derived from the individual and family domains by including school experiences, sociodemographic background and family variables (task fulfilment, role performance, communication, affective expression, connectedness, control behaviour and values and standards) in the analysis. These thematic analyses will establish which experiences are specific to school dropouts with mental health problems, which are specific to school dropouts without mental health problems, and which to students enrolled in regular secondary education.

## Methods/Design

The study design consists of a nationwide case control study. The projected period of study are the 2010/2011 and 2011/2012 academic years. The study protocol has been revised and fully approved by the national research ethics committee ("Comité National d'Ethique de Recherche" CNER, approval N° 201009/07 version 1.1) and it has been notified to the national commission for data protection ("Commission Nationale pour la Protection des Données" CNPD).

### Case definition

In Luxembourg, the ministry operates a centralized management system for secondary education that allows identifying "school dropouts", i.e. students who leave school during the academic year or at year-end without any graduation. The definition applies for students who definitely left the educational system and have no specific occupation at all, but also for students who left school and began to work, or registered with an employment program. It also includes so-called "temporary" dropouts, who reregister with the educational system after some period of absenteeism (more than 3 months). For this research, temporary dropouts will be eligible as we tend to interview the student as soon as possible after he/she had dropped out.

### Control definition

The control group will be chosen from the same source and composed by students who are enrolled in regular secondary education and who attend classes. They will be individually matched for age, gender, school and educational grade.

### Inclusion criteria

Eligible participants are aged 16-25 years, residing in Luxembourg and enrolled in a national public secondary school. Since data will be collected in face-to-face interviews, participants must be reachable (students in acute hospitalization or under arrest are not eligible). They also need to understand either German or French, which are the languages of self-report questionnaires. An informed consent must be signed by the student and his/her legal guardian if he/she has not attained full age.

### Recruitment procedure

**Figure 1:**
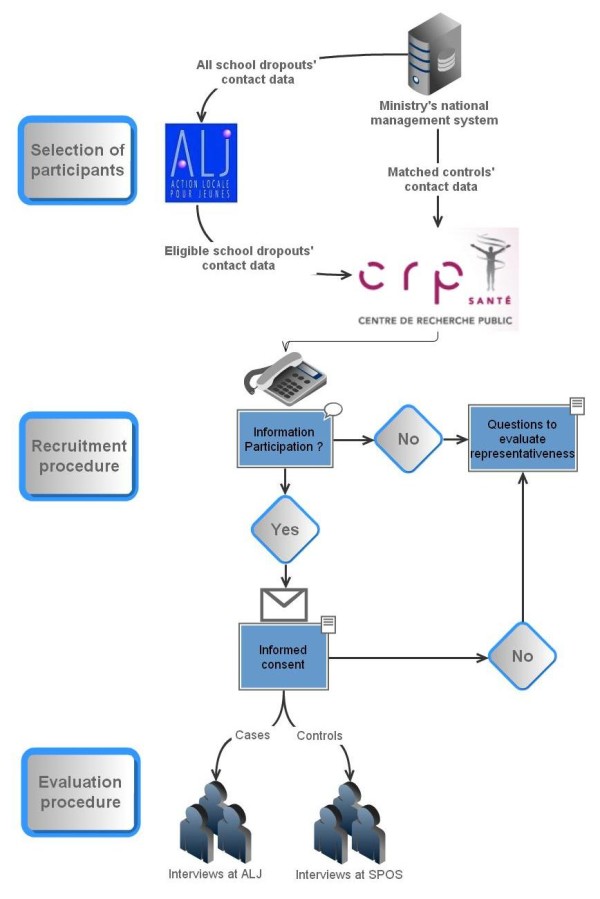
**Stages of field research**. Figure 1 gives an overview of field research stages, beginning with the selection of eligible participants realised in collaboration with the ministry and "Action Locale pour Jeunes" (ALJ), their recruitment and finally the evaluation procedure, which consists of face-to-face interviews.

A request will be submitted to the ministry of education (MENFP) to get contact data of eligible participants. As shown in Figure 1, the recruitment procedure slightly differs for cases and controls.

Figure 1: Stages of field research

The recruitment of school dropouts will be incident and realised in collaboration with a service operated by the ministry and called "Action Locale pour Jeunes" (ALJ). Every month, the ALJ receives a listing of all school dropouts who they contact in order to find out about their current occupation. If needed, they provide academic or vocational support.

As we expect a major denial of participation, all eligible dropouts listed in a monthly record will be systematically contacted.

A preliminary telephone contact provides information on the study (objectives, procedure, expected benefits) and asks for participation. If the student is interested in participating, an appointment for the interview is immediately set up and informed consent will be sent. It should be noted that information avoids deterrent terms such as "psychiatric" or "mental" and rather refers to "personal wellbeing". Meetings with cases will be organised in the regional offices of ALJ as we consider it an uncommitted, well accepted site.

The recruitment of controls will be subsequent to the participation of dropouts. There will be one control per dropout who participated in the study. Expecting a major denial of participation, we will ask the ministry to get a selection of 5 matched controls per case. Among these 5 controls, selection will be randomised.

The recruitment and evaluation procedures will be same than for cases (cf. Figure 1), but interviews will take place at school and outside of school hours.

### Sample size calculation

Considering an overall lack of prevalence rates of mental disorders in Luxembourg, we refer to the European Study of the Epidemiology of Mental Disorders [4] to assume a prevalence rate of 13.7% for any psychiatric disorder in the age group 18-24 years. Previous studies have reported a significant association between psychiatric disorders and subsequent school dropout by identifying odds ratios ranging from 1.0 to 5.7 depending on the disorder category [11,13,14]. To demonstrate that young adults with a psychiatric diagnosis compared with young adults without any psychiatric diagnosis have an odds ratio (OR) of at least 2.0 for dropping out of school, with alpha error set at 0.05 and statistical power of 80%, 146 participants per group will be needed.

### Measures

During a face-to-face interview, a trained psychologist will investigate about socio-demographic background, school experiences, mental health and family life by providing the self-report questionnaires and (semi)structured interviews listed below.

#### 1. Demographics and schooling

We will use a semi-structured non standardised interview to question on nationality/ies, native language/s, household composition, educational attainment, school engagement, performance and aspirations, behaviour in class and employment status. Most questions are derived from the "Health Behaviour in School-aged Children" [28] questionnaire used in the 2009/2010 survey.

#### 2. Socioeconomic status

The "Family Affluence Scale" (FAS) is a questionnaire developed by the WHO and used in the HBSC surveys. Questions are easy to answer and evaluate material conditions of the participant's household (car ownership, bedroom occupancy, holidays and computers). The summed score of these 4 items is recoded to give values of low, middle and high family affluence [29]. Advantages of this measure are the low percentage of missing responses and the very economical time management.

#### 3. Mental health

Data will be collected using a composite of measures.

Depending on the age of the participant, we will choose a self-report form from the Achenbach System for Empirically Based Assessment (ASEBA). Students who haven't reached their majority on the date of the meeting will answer the Youth Self-Report YSR [30] which is adapted for adolescents aged 11-18 years. The 112 items are worded in the first person and the response format is 0 (not true), 1 (somewhat or sometimes true) or 2 (very true or often true). The YSR can be scored on a total problem score and on eight syndrome scales: withdrawn, somatic complaints, anxious/depressed (together constituting the Internalizing Scale); rule-breaking behaviour, aggressive behaviour (together constituting the Externalizing Scale); social problems, thought problems and attention problems [31].

From 18 years on, students will answer the Adult Self-Report ASR [32], which covers the age span 18-59 years. As for the YSR, the 126 items can be scored on eight syndromes scales: withdrawn, anxious/depressed (together constituting the Internalizing Scale); intrusive behaviour, rule-breaking behaviour, aggressive behaviour (together constituting the Externalizing Scale); somatic complaints, thought problems and attention problems [31].

Furthermore, diagnoses of psychiatric disorders according to criteria of DSM IV or ICD 10 will be obtained from the latest official version of the MINI International Neuropsychiatric Interview [33]. Disorders are explored at a current, past and/or lifetime timeframe and cover 1) anxiety disorders (generalized anxiety disorder, panic disorder, agoraphobia, social phobia, obsessive compulsive disorder, posttraumatic stress disorder), 2) eating disorders (anorexia nervosa, bulimia nervosa), 3) mood disorders (major depressive episode, manic episode, hypomanic episode, bipolar disorder), 4) suicidality, 5) alcohol and drug dependence/abuse, 6) psychotic disorders and 7) antisocial personality disorder.

The section on dependence/abuse disorders will be completed by exploring internet use, a more recent pattern of addictive behaviour. Therefore, we will refer to the Internet Addiction Test (IAT) [34] which is based on the DSM IV criteria of pathological gambling and alcohol abuse. It consists of 20 items rated on a 5-point Likert scale (rarely, occasionally, frequently, often, always). The summed score provides a measure of problematic use.

#### 4. Family life

"Familienbögen" is a German self-report questionnaire exploring the following dimensions of the family system: task fulfilment, role performance, communication, affective expression, connectedness, control behaviour as well as values and standards. The 40 items are rated on a 4-point response scale (strongly agree, agree, disagree, strongly disagree) [35].

Data management will be done using Capture System.

### Linguistic validations

Considering two major trends in the multilinguistic culture of Luxembourg, we will provide German and French translations of all questionnaires. There are official German and French versions for the ASEBA forms [36] and the MINI, as well as a validated French translation for the IAT [37]. Questionnaires on family life and internet use were translated according to the linguistic validation procedure (forward & backward translations, testing).

### Statistical analysis

To evaluate the representativeness of the school dropouts included in the study, a comparative analysis will be conducted on age, gender, educational grade, grade repetition, nationality, household composition and employment status.

Results will be expressed as mean and standard deviation (SD) for quantitative measurements and as frequency tables for categorical findings. The association between two continuous variables will be assessed by the correlation coefficient. Matched groups means values will be compared by two-way analysis of variance (or paired Student t-test) while the symmetry test (or McNemar Test) will be used for paired proportions. To identify variables associated with school dropout, we will conduct a conditional logistic regression analysis for matched pairs' data with school dropout as the outcome and psychiatric disorders, socioeconomic background and family variables as predictors of interest. The approach used to model psychiatric disorders within cases and controls with risk factors and/or prognostic factors will consist in a stepwise descending regression modeling technique starting from the full model in which all potentially and clinically important variables are included. Statistical control factors will concern demographic variables and school experiences. Comparison of embedded models will be done with the likelihood ratio test. Results will be considered as statistically significant at the 5% critical level (p < 0.05). All calculations will be carried out using the SAS^® ^System (Version 9.2 for Windows) statistical package and SPSSInc PASW Statistics 18.

### Feasibility study

A feasibility study including 10 participants per group will evaluate the recruiting procedure, the interview sessions and the constitution of the test battery. This preliminary stage will take place in exactly the same conditions as the future study.

## Discussion

In the light of the individual, social and economic burden of school dropout, policies to promote adolescents' participation in education should be considered as an investment in human capital. At the European level, stakeholders are invited to take action on mental health in youth and education by training professionals and implementing early intervention programs throughout the educational system to prevent abuse, bullying, violence and social exclusion [38]. As public health strategies should focus on a certain number of distinct pathways rather than on non-specific, generalized effects of poor mental health on educational attainment [13], dropout research should be based on highly reliable, specific predictor and outcome variables and sophisticated multivariable analyses [39]. However, due to deficiencies either in measurements (self-report measures, recall bias), study design (retrospective, no comparison group) or sampling (sample size, group definition), current dropout research seems largely insufficient to meet these conceptual and methodological requirements.

Through its prospective case control design and its comprehensive measures of school and non-school (mental health, socioeconomic background and family functioning) correlates of dropout, this study hopes to improve on previous research.

The design is nevertheless limited by the use of end-point measures that will not allow the sequence of events to be established. Since no measures of exposure are available before the subject left school, it is possible that mental health and family functioning have been influenced by the dropout.

Data collection will be optimised by providing a combination of self-report forms and structured interviews during a face-to-face meeting with a trained psychologist and the participant. This setting may allow to increase the accuracy of data by reducing participants' inhibition as well as response patterns based on social acceptance.

One possible issue regarding measurements may be that we will not consider data from teacher or parent reports. Although it seems customary to collect data from multiple sources, several studies concluded on a significant divergence between the sources [40-42]. In fact, Roberts [42], recorded considerable differences in parent and child ratings across ethnic groups: minority parents reported fewer psychosocial problems, whereas no differences were observed among the children. Considering these findings in the multicultural context of Luxembourg, we suppose that reliance on adolescent reports may be less problematic than the use of parent reports. We also assume that, a parent or teacher who rates a student's functioning, is more likely to focus on behavioural aspects because they are more obvious. The use of multiple sources may thus overestimate behavioural problems and underestimate emotional ones.

## List of abbreviations used

PPSM: Prévention et Promotion de la Santé Mentale; OECD: Organisation for Economic Co-operation and Development; MENFP: Ministère de l'Eduction Nationale et de la Formation Professionnelle; DSM IV: Diagnostic and Statistical Manual of Mental Disorders IV; ADHD: Attention Deficit Hyperactivity Disorder; SES: socioeconomic status; CNER: Comité National d'Ethique de Recherche; CNPD: Commission Nationale pour la Protection des Données; ALJ: Action Locale pour Jeunes; SPOS: Service de Psychologie et d'Orientation Scolaires; HBSC: Health Behaviour in School-aged Children; FAS: Family Affluence Scale; WHO: World Health Organization; ASEBA: Achenbach System for Empirically Based Assessment; YSR: Youth Self-Report; ASR: Adult Self-Report; ICD 10: International Classification of Diseases 10; MINI: MINI-International Neuropsychiatric Interview; IAT: Internet Addiction Test.

## Competing interests

The authors declare that they have no competing interests.

## Authors' contributions

LFH and MG conceptually developed the idea for this research. All authors contributed to the development of the study protocol, the conception of methods and the selection of diagnostic instruments. VB did the sample size calculation. PE did the writing of the manuscript. MA, SC and MLL revised and edited the draft. All authors have read the manuscript, made contributions and approved the final text.

## Pre-publication history

The pre-publication history for this paper can be accessed here:

http://www.biomedcentral.com/1471-2458/11/555/prepub
